# Evaluation of Salmon (*Salmo salar*) and Rainbow Trout (*Oncorhynchus mykiss*) pin bones using textural analysis and micro X-ray computational tomography

**DOI:** 10.1007/s13197-019-03803-9

**Published:** 2019-05-23

**Authors:** Schroeder Sarah, Savage Anne, Grigor John, Sturrock Keith, Cassidy Philip, Töpfl Stefan, D. Wilkin Jonathan

**Affiliations:** 1 University of Applied Science Osnabrück, Albrechtstraße 30, 49076 Osnabrück, Germany; 2 Food and Drink Division, School of Applied Science, Abertay University, Bell Street, Dundee, DD1 1HG Scotland, UK; 3 Science Division, School of Applied Science, Abertay University, Bell Street, Dundee, DD1 1HG Scotland, UK; 4 Dawnfresh Seafoods Ltd, Bothwell Park Industrial Estate, Uddingston, Glasgow G71 6LS UK

**Keywords:** Pin bones, Deboning, Trout, Salmon, Texture analysis, CT scan

## Abstract

Industrially, common problems arise with the deboning pin bone process, where Atlantic Salmon (*Salmo salar*) and Rainbow Trout (*Oncorhynchus mykiss*) fillets, *post rigor*, are subjected to a pulling process to remove the pin bones from the fillet. This study measured the length of pin bones from two species of fish and two different industrial graded weights, and then used a texture analyser and µCT X-ray to measure the pulling force, break point and volume of the pin bones of both species of fish. Results showed that salmon pin bones required significantly higher pulling force to remove pin bones from the fish fillet when compared with Trout pin bones. Interestingly Trout pin bones were significantly longer and stronger than Salmon pin bones, but had significantly lower volume. This research has progressed the issues surrounding pin boning industrially, however, more studies are required in order to understand if these differences affect the overall deboning pin bone process.

**Electronic supplementary material:**

The online version of this article (10.1007/s13197-019-03803-9) contains supplementary material, which is available to authorized users.

## Introduction

Almost all fish products sold on the market in UK are prepared for direct cooking, which means that they are processed “pin-bone free”. Deboning is therefore an important step within the manufacturing process of fish, which includes Atlantic salmon (*Salmo salar*) and Rainbow trout (*Oncorhynchus mykiss*). A modern food industry requires the removal of all physical hazards within food products; pin bones are considered a foreign body when inadequately removed and are a common source of customer complaints (Mery et al. 2011; Borderías and Sánchez-Alonso 2011). These ‘missed’ pin-bones can cause a health hazard to consumers through injuries in the mouth and oesophagus (if accidently ingested) and a food safety hazard if piecing the film of the product, thus resulting in microbial growth (Balaban et al. 2015). Atlantic Salmon and Rainbow Trout are manufactured/processed the same way in industry, but in industry difficulties in de-pin-boning trout can occur during processing, when compared to salmon.

Pin bones are found in the rib area of the fish and occur only in fish from the superorder of Teleostei (Hoar 1976). The bones extend from the spine into the muscle tissue but are not directly connected to the spine, where they develop in the intermuscular border on both sides of the fillet and are attached to the ligaments (Sahu et al. 2014). From a biological point of view, pin bones should not be classified as bones seeing that they are formed when ligaments calcify and do not contain bone marrow, and are sometimes referred to as “false ribs” (Sahu et al. 2012). They are light and thin but relatively large (Sahu et al. 2014; Mathiassen et al. 2012) and are either a Y shape or straight (Sahu et al. 2012). Interestingly enzyme profiles seem to differ between species, where cod and salmon enzymes within the connective tissue matrix metalloproteases varied greatly between the two species (Vuong, et al. 2017).

Deboning takes place 4–6 days after the fish has been slaughtered; this is due to *rigor mortis* phase, after death in which the fillet becomes inflexible (Thielemann et al. 2007; Morison 2004). The pin bones cannot be pulled out during *rigor mortis* without breaking or damaging the fillet due to it being attached to tendons in the muscle (Mathiassen et al. 2012). Some pin bones still tend to break after *rigor mortis* or may be difficult to remove as they have an exceptionally strong attachment to the tendons of the muscle (Wang et al. 2015).

Balaban et al. (2015) showed that the size of fish generally affected the pulling distance and peak pulling force when removing bones. Similarly, they measured the differences between locations of the pin bone and saw variation although not significant. During this study, variation in the pulling force of pin bones was observed and the authors suggested that it was due to the sampling regime and moisture content of the pin bones, although no further work was included. Our previous study reported the use of calcium chloride and collagenase treatments for the successful reduction of pulling force associated with pin bones from salmon and trout (Schroeder et al. 2018). Conflicting literature showed moisture contents of salmon flesh ranged from 77.06 to 80.89% (Dempson et al. 2004), whereas Craft et al. (2016) showed considerably lower moisture content for trout (59.5–63.7%). In essence the results suggests that there is a correlation between length of pin bone and pulling force, where the longer the pin bone the more force required to remove it (Balaban et al. 2015).

## Materials and methods

### Materials

Fresh fish samples of Atlantic salmon (*Salmo salar*) and Rainbow trout (*Oncorhynchus mykiss*) were obtained from Dawnfresh Seafoods Ltd. and were classified into two groups for analysis these were 3–4 kg and 4–5 kg weight range, all samples were female triploids for trout, and females for salmon. Each farmed fish sample was filleted but not deboned and was provided *post rigor* and analysed fewer than 6 days from harvest. All samples were quality assessed visually and analysed in triplicate.

### Pin bone pulling force

A TA.XT plus texture analyser (Stable Microsystems, Surry UK) with a friction rig attachment and 50 kg load cell was used to measure the force required to remove pin bones from the fish fillet. The test mode was Tension, with a pre-test speed of 2 mm/s, test speed 1 mm/s and post-test speed at 10 mm/s. The distance travelled stopped measuring at 20 mm. Each pin bone was removed and total force needed to pull the bones out of the fish fillet were recorded and labelled, 29 pin bones recorded for salmon and 31 pin bones recorded for trout [labelled from the tail (31/29) all the way to the neck (1)]. Pin bones were selected as 29 for salmon and 31 for trout based on previous work, where the authors (Schroeder et al. 2018) showed that this was a natural variance between the species of fish (Balaban et al. 2015).

### Breaking point

On separate fish fillets (4–5 k), the pin bones were pulled out of the fresh fish fillets by hand using pliers and measured individually using a ruler, once dry (24 h at room temperature). Following a similar method to Chambi and Grosso (2006) with slight modifications, a texture analyser (TA.XT TA.XT plus texture analyser (Stable Microsystems, Surry UK)) was used with a 5 kg cell load with tensile grips fixed to the top and the bottom of the sample (in this case) bone. The grip separation and crosshead speed used initially were 30 mm and 1 mm/s, respectively, and the grips moved apart during the testing and recorded each samples observed break point. The texture analyser measured the force required to break the salmon and trout bones, which were measured in triplicate.

### CT scanning: bone angle and volume

An X-TEK CT-Scanner HMX 225 fitted with a Nikon detector was employed. A 0.25 mm aluminium filter was attached to reduce the noise during the scanning and the settings were adjusted as follows: set at 75 kV and 65 µA and sample scanning took approximately 90 min. Following scanning the generated images were constructed into a 3D volume file using CTAgent software (Nikon).

In order to prepare fillet samples of an appropriate size to fit within the µCT scanner, whole (headless and filleted but not pin boned) fish (4–5 k) were divided in half at pin bone 20 and any excess parts were removed. It was not possible to control the temperature of the sample during the scanning procedure but the samples were contained in plastic to slow down drying out of the samples during the procedure. Using VG Studio Max™ 3.1 each volume file was cleaned graphically to remove any artefacts not associated with the fish fillet and then the flesh around the pin bones made transparent so that only the pin bones were visible (this was based on the density differences between the sample flesh and bone). The volume of each pin bone was measured using VG Studio Max™ 3.1 and are expressed as mm^3^.

### Statistics

Pin bone length were analysed by a Wilcoxon Sign Rank Test in IBM SPSS ver 24. Each species was analysed separately and the main factor was size of fish. There was one null hypothesis of interest; size of fish had no effect of the length of pin bones in both species. For volume of the bones, a Paired Samples T-Test was used. Species were compared and the main factor was specie. There was one null hypothesis of interest; species of fish had no effect on the volume of pin bones. For the force required to remove pin bones a nested ANOVA was used. Each species was analysed separately and the main factor was size of fish. Pin bones number was were nested inside fish. There were two null hypotheses of interest, (a) size of fish had no effect of the force required to extract a pin bone (b) pin bone location had no effect on force required to extract the pin bone. Lastly, a Paired Samples T-Test was used to compare the differences between break point of the bones; however, the data was transformed due to a positive skewed distribution prior to statistical analysis. There was one null hypothesis of interest; species of fish had no effect on the breakpoint of the pin bone.

## Results and discussion

### Pin bone length analysis

The median of a salmon 3–4 kg was 38 cm; 40 cm for salmon 4–5 kg; and for trout, 3–4 kg was 37 cm, 45 cm for trout 4–5 kg. Comparing pin bone length between species of fish shows similar profiles of pin bone length when compared between species, although Trout pin bones are longer at pin bone 14–31 than Salmon pin bones, which are larger at pin bone 4–13. Salmon tended to have smaller bones in the neck area and tail area, but both observed lower bone sizes between species and weight of fish. The distribution of bone length went from smaller bones in the neck and tail (the lowest measurement was 14 mm, 18 mm, 15 mm and 20 mm, salmon 3–4 k and 4–5 k, and trout 3–4 k and 4–5 k, respectively). Trout 4–5 k (55 mm) samples showed longer bone lengths than Trout 3–4 kg (50 mm), but salmon had no obvious differences in lengths between weights (both sizes maximum bone length was 50 mm).

**Fig. 1:**
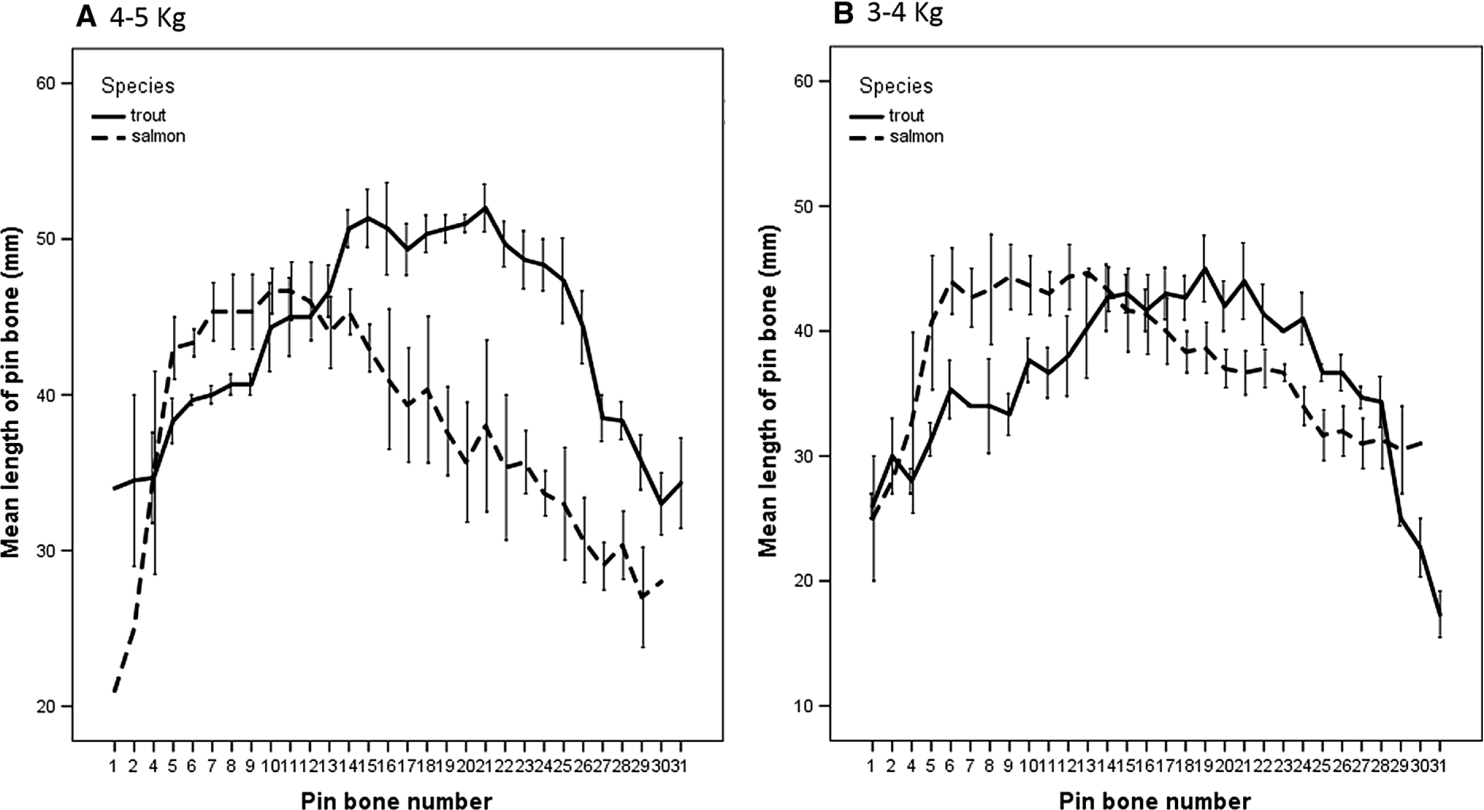
The size of pin bones (mm) along the length of salmon and trout. The data represent average + standard error of three fish of similar weight and from each species. **a** (4–5 kg) **b** 3–4 kg

This study examined the variation in pin bone length by location within salmon and trout fish and examined whether there was species variation. A mixed effect model was produced in SPSS v24 with pin bone number and species (trout or salmon) as fixed factors. The experiment comprised three individual fish of each species and variation between replicate fish was accounted for by treating the fish ID as a random factor. For fish between 4 and 5 kg (Fig. 1a), the length of pin bones varied significantly (F(29,104) = 17.72; *p* = 1.8 × 10^−28^) but salmon pin bones were on average longer than trout pin bones between approximately pin bone 4 and 10 (Fig. 1a) but after this point, trout bones were longer than salmon bones. Overall, it is not possible to state which species had the longer pin bones because this depended on the location within the fish i.e. there was an interaction between pin bone number and species (pin bone location * species: F(28, 104) = 9.18; *p* = 1.7 × 10^−17^). The assumptions of the mixed effect model were satisfied thereby validating this result; the residuals were normally distributed according to a Shapiro Wilks test (W (167) = 0.988; *p* = 0.151) and the normal Q–Q plot of residuals was linear (see Online Appendix). A plot of the residuals versus the predicted values produced a random pattern indicating that the magnitudes of the residuals were independence of the predicted value of the pulling force. Finally, there was a linear relationship between the model’s predicted value for the pulling force and the measured pulling force. A similar result was obtained for the smaller fish (3–4 kg—Fig. 1b). Which species had the longer bone depended on the position of the bone being measured with the salmon having the longer bones between position 5 and 12 but beyond this point, the trout having the longer bones. In other words for the pin bone length, there was a significant interaction between the pin bone position and species (F(28,106) = 4.55; *p* = 6.5 × 10^−8^). The magnitude of the differences in small fish were not as great as the differences in larger fish but again, as in larger fish, the pin bone length in smaller fish varied significantly according to its position F(29.106) = 14.2; *p* = 6.2 × 10^−25^). The assumptions of the model for the smaller fish were partially satisfied. Although the histogram of the residuals was approximately symmetrical, there were a few outliers in the normal QQ plot and the Shapiro Wilks test indicated deviation from normality (W(169) = 0.974; *p* = 0.003). However, the plot of the residuals versus predicted values indicated that they were independent and the bone lengths predicted by the model and the measured lengths varied linearly (see Online Appendix).

Large salmon pin bones were defined by Mery et al. (2011) as being larger than 12 mm in length, small were considered below 8 mm in length and medium pin bones were between 8 and 12 mm in length. Balaban et al. (2015) showed salmon pin bone lengths to range from 30 to 55 mm. Previous studies discussed that fish pin bones for trout ranged from 14 to 47 mm in length, thus larger than salmon (Mery et al. 2011). The results presented here concur with previous studies, where trout bones generally are larger than salmon bones. The bones from both species in this study were considerably larger than in these previous studies (salmon 20–47 mm; trout 30–47 mm), however, the weight of fillets tested in the earlier studies were considerably lower than those tested within this study. Due to the size of fish and differences in the length of pin bones, pin bones of various lengths were further investigated to examine differences in volume sizes, pulling force and break point of the pin bones.

### Pin bone volume

**Fig. 2:**
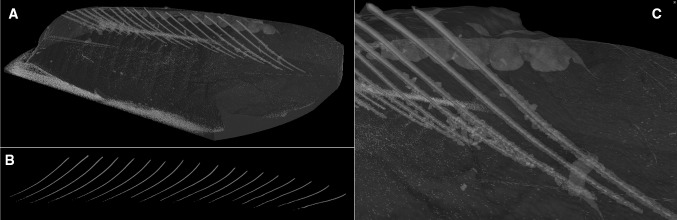
A typical sample captured by a micro X-ray CT scanner (trout 4–5 k) for volume analysis. **a** A section of a trout fillet with bone highlighted, **b** bones in situ with flesh removed, **c** closer examination of the bones within the flesh of the fish

Figure 2 shows a typical scan of the fish fillet with the µCT X-Ray scanner and Table 1 shows the mean pin bone volumes (mm^3^) compared between the two species of fish, note that for this experiment, only Salmon and Trout 4–5 k fish fillets were analysed. The fish were cut in two separate pieces at pin bone 20, for this experiment only the neck and belly area were analysed for volume. Trout bin pones showed a smaller volume than salmon pin bones and showed significant differences through Paired Samples (t(90) = 17.506, *p* < 0.000). The difference between the pairs of pin bones were normally distributed (Shapiro–Wilks(91) = 0.986; *p* = 0.448) so assumption of the Paired sample t-test is satisfied. The variance in Salmon bone volume was larger than the variance in Trout bones volumes. Typically 4–5 k Trout fillet pin bones were shown to be 5–8 mm^3^ and 4–5 k Salmon fillet pin bones were 8–12 mm^3^. The lowest measurement of pin bone volume for salmon was 1.47 mm^3^ and 0.17 mm^3^ for trout, and trout had lower maximum pin bone volume (11.07 mm^3^) than salmon (16.85 mm^3^). The pin bone volume suggests that, although trout pin bones are longer, as seen previously, they have less volume associated with them, which may mean that they break more easily during filleting. No past data has been published which measured Trout and Salmon pin bone volumes nor compared them with each other. The results suggest that pin bones from Trout are longer and thinner than Salmon pin bones in 4–5 k graded fish fillets, which may cause issues in the future if the same method of removal is used in industry for both species. It is the recommendation of this study, that further work is conducted on the seasonality and yearly changes in this post-harvest studies to understand if this phenomenon is solely a sample and timing issue; and not a species difference. Then the design of industrial species-specific deboning machines can remove the majority of pin bones within the fish products.

**Table 1:** Shows the mean pin bone volumes (mm^3^) of 4–5 k salmon and trout fish fillets, with standard deviation

Salmon mean pin bone volume (mm^3^)	Trout mean pin bone volume (mm^3^)
9.38 ± 3.94	6.32 ± 2.62

### Pulling force analysis

**Fig. 3:**
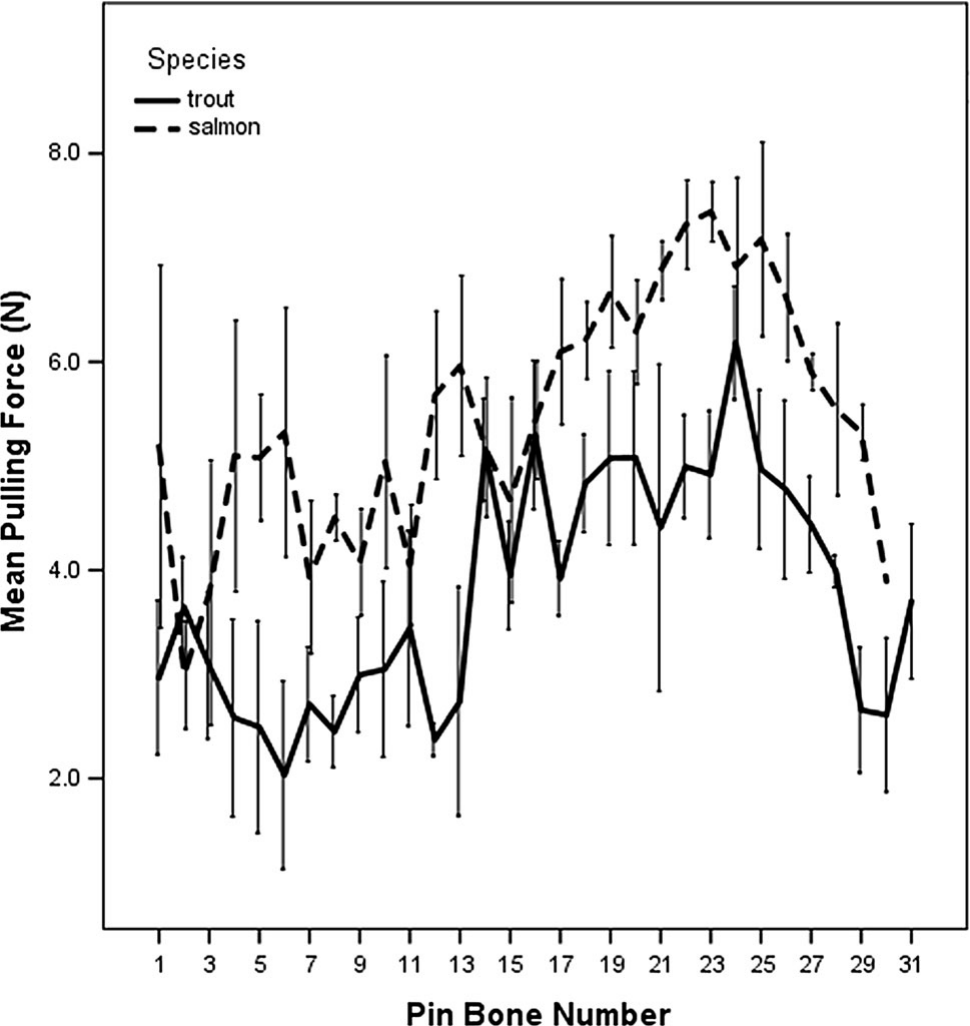
The pulling force required to remove pin bones along the length of salmon and trout (N). The data represent average + standard error of three fish of similar weight from each species

The pulling force (N) required to remove pin bones from Salmon and trout (4–5 kg fillets) are shown in Fig. 3. A and C show the differences between species and pin bone pulling force removal, where salmon generally had the higher force required to remove the pin bones. Trout however, showed higher forces required from the belly of the fish to the tail, whereas Salmon showed similarly to Salmon but less pronounced data. The highest force was from Salmon 7.44 N (758.67 g) and 6.18 N (630.18 g) for Trout, the lowest was 2.03 N (207.00 g) for Trout and the lowest pulling force for Salmon was 2.99 N (304.90 g). The mean Salmon pulling force was 5.41 ± 1.71 N and 3.79 ± 1.52 N for Trout fillets pulling force.

The aim of this study was to see if either species or pin bone location influenced the pulling force (N) required to remove a pin bone. A mixed effect model was produced in SPSS v24 with pin bone number and species (trout or salmon) as fixed factors and individual fish number as a random factor. The experiment comprised three individual fish of each species and variation between replicate fish was accounted for by treating the fish ID as a random factor. There was a main effect of species (F(1,4.04) = 9.17; *p* < 0.038) on pulling force with salmon requiring more force on average than trout to remove a pin bone from the same location. The pulling force required to remove a pin bone depended on its location for both species with bones between the centre and tail requiring more force than the head region (F(30,113) = 5.01; *p* = 1.1 × 10^−10^). There was no interaction between pin bone location and species (F(29,113) = 1.120; *p* = 0.248) so although the salmon pin bones required more force on average than the trout bones, the pattern in the variation of force required along the length of the fish was similar for both species. The assumptions of the mixed effect model were satisfied thereby validating this result; the residuals were normally distributed according to a Shapiro Wilks test (W (178) = 0.996; *p* = 0.904) and the normal Q–Q plot of residuals was linear (see Online Appendix). A plot of the residuals versus the predicted values produced a random pattern indicating that the magnitudes of the residuals were independence of the predicted value of the pulling force. Finally, there was a linear relationship between the model’s predicted value for the pulling force and the measured pulling force.

This work followed similar trends as previously reported by Balaban et al. (2015), where larger pin bones required higher forces to be removed from the fillet than smaller pin bones. However previous studies showed larger forces required to pull pin bones from salmon fillets from pin bone 1 through to 30 (Balaban et al. 2015), this study showed some pin bones required less force (below 3 N). Balaban et al. (2015) did explain that more samples were required to further understand the pulling force on bone removal, however in this study some of these pin bones may have been affected by the filleting process, all samples were pre-fileted using industrial fileting process prior to analysis, whereas Balaban et al. (2015) study the fish was hand filleted. The filleting process may have adversely affected those pin bones within the neck area thus lowering the pulling force. Further work is required to understand the whole filleting process from de-heading to spine removal and its effects on pin bone pulling force. Clearly, Salmon required significantly more pulling force than Trout in this testing period, again as post-harvest investigations more information is required on the seasonality as well as harvest year to fully understand the effects of filleting and pulling force the removal of pin bones from these two species of fish. Our previous report has shown, like this report that salmon bones required.

For both trout and salmon, pin bone location, significantly influences the force required for extraction and removal of pin bones from large trout requires less force than removal of the corresponding pin-bone from small trout. Size of fish however does not influence ease of pin bone removal from salmon. Salmon pin-bones near the tail require the most force for removal but extraction force required gradually declines towards the head. For trout however, the pin bones in the centre of the fish seem to require the most force for removal.

### Breaking point analysis

**Fig. 4:**
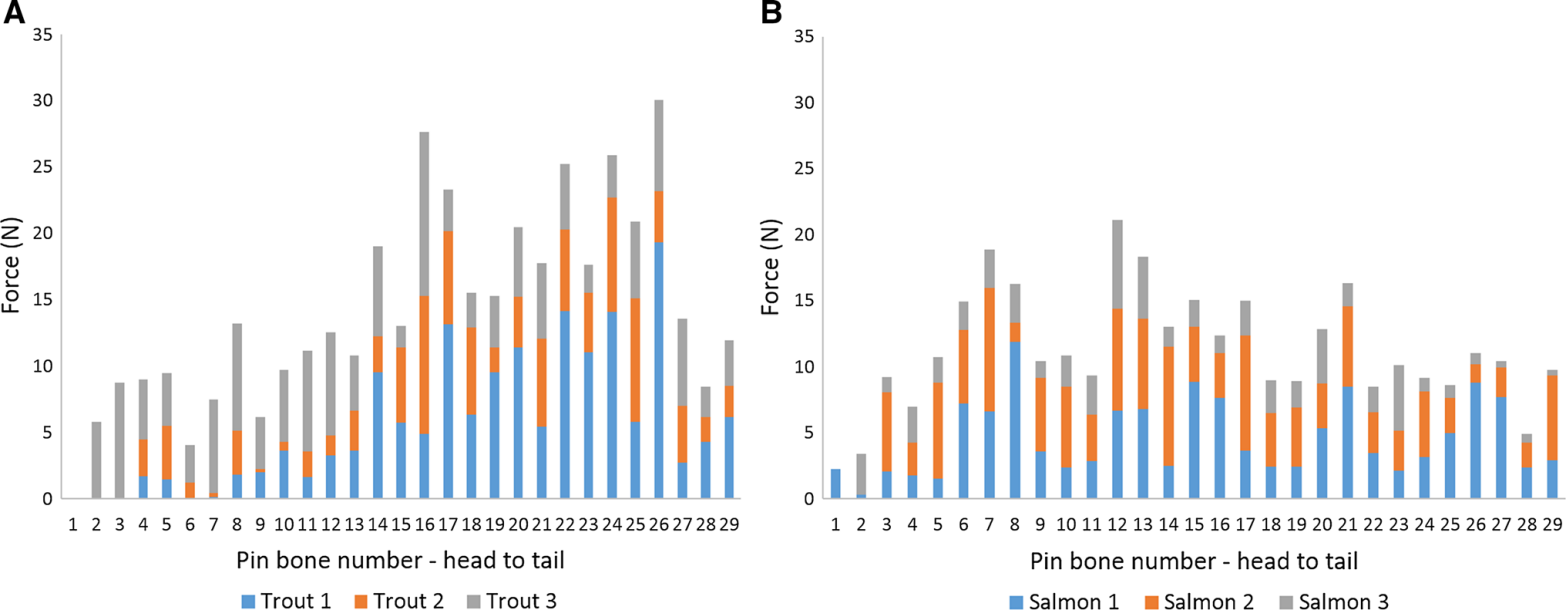
Variation in the break point of salmon (**a**) and trout (**b**) pin bones. Error bars are standard error n = 3 fish for each species (4–5 k)

Figure 4a shows the break point of the bones from 4 to 5 kg salmon and Fig. 4b for trout fillets, where the highest recorded break point was from Trout 19.34 N (1972.13 g), Salmon was considerably lower 11.92 N (1215.50 g). The lowest recorded break point for Salmon was 0.35 N (35.69 g) and 0.10 N (10.20 g) for Trout. The overall pin bone mean breaking force were 3.91 ± 2.56 N and 5.18 ± 3.65 N for break point for Salmon and Trout respectively.

The data was analysed using a Paired sample t-test to investigate the effects of species and pin bone number on breaking point. There was a significant difference between species and break point (t(163) = − 19.984, *p* < 0.000). The difference between the species and breakpoint were normally distributed (Shapiro–Wilks(164) = 0.992; *p* = 0.470) so assumption of the Paired sample t-test is satisfied.

Balaban et al. (2015) showed higher force in breaking the bones compared with the method specified here, which could have been due to methodological approaches. In this paper, the experiments were carried out using A/MTG Mini Tensile Grips, whereas previous studies used fine sandpaper sandwiched between tensile grips. Interestingly within this study, Trout bones were stronger than Salmon bones, but required less force to remove, were longer and had a smaller volume. The results on the force required to remove pin bones from the fillet, the break point of pin bones and the length of pin bones are inconclusive to determine the reason as to why trout bones are more of a problem than salmon bones. Generally, the hypothesis was if the trout bones were more troublesome, one would expect this to form three categories, differences in the size of pin bones, the required pulling force and the break point, however in this study, salmon had higher pulling force to remove the bones; trout had larger pin bone size and were stronger. However, the volume of the Trout pin bones compared with Salmon pin bones suggests that perhaps this is one of the reasons for the difficulties experienced during industrial processing. More work is being carried out on the removal of trout and salmon pin bones, including our own work (Schroeder et al. 2018), but also the use of ultrasound to lower the pin bone force (Skjelvareid et al. 2017).

## Conclusion

Both fish meet different requirements for the deboning process, which is the reason why there are so many difficulties in the bone removal process. In conclusion, it is recommend that in order to provide assistance to the deboning process, all variables are considered to fully understand the effect of species, size of fish and other variables on extraction pin bone from salmon and trout, before machines for pin deboning are developed or further validated.

## Electronic supplementary material

Below is the link to the electronic supplementary material.

Supplementary material 1 (DOCX 145 kb)
