# Supplementary figures and images for: Evaluation of Salmon (Salmo salar) and Rainbow Trout (Oncorhynchus mykiss) pin bones using textural analysis and micro X-ray computational tomography
Source: J Food Sci Technol. 2019 May 23;56(7):3313–9. doi: 10.1007/s13197-019-03803-9 (PMC6582037; doi:10.1007/s13197-019-03803-9)

Appendix


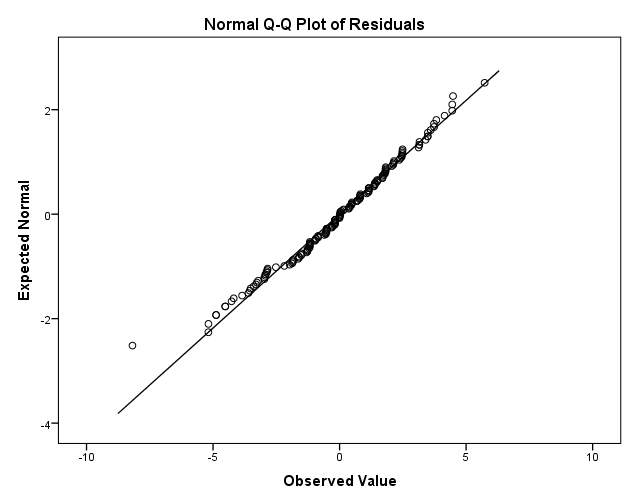


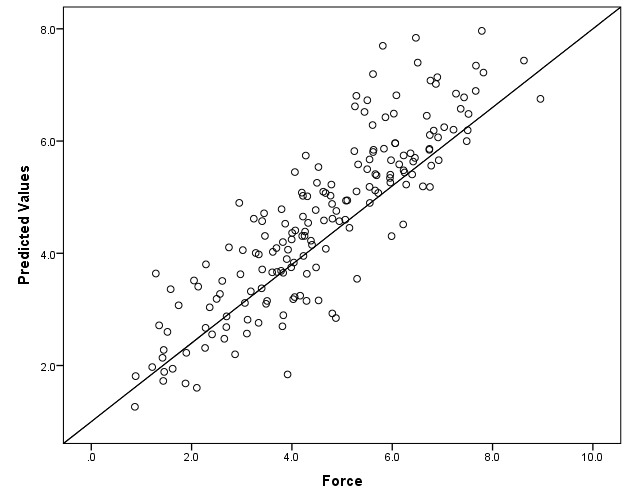


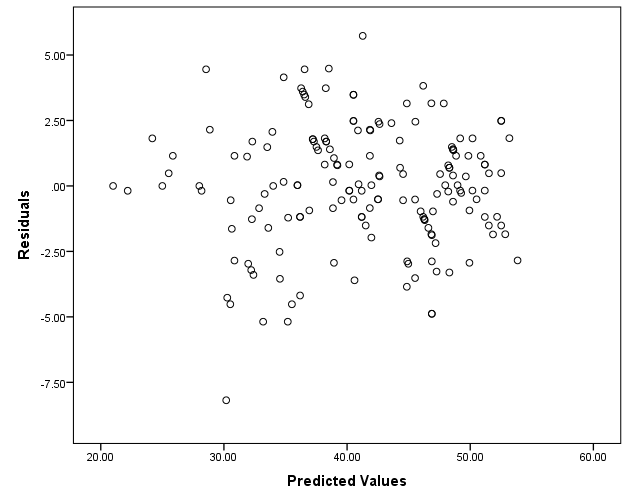


Appendix for Figure 2(b)


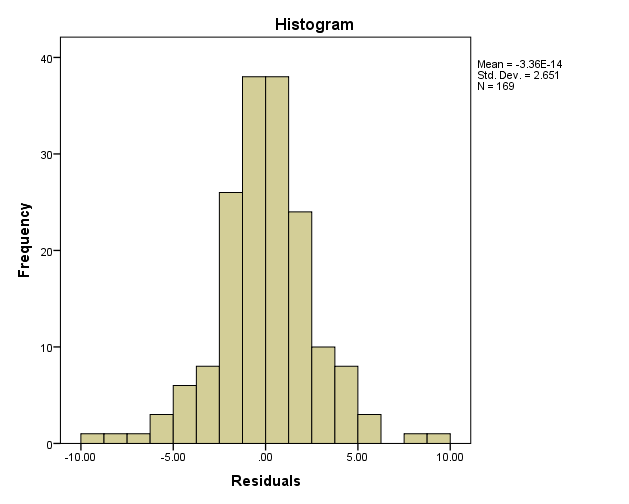


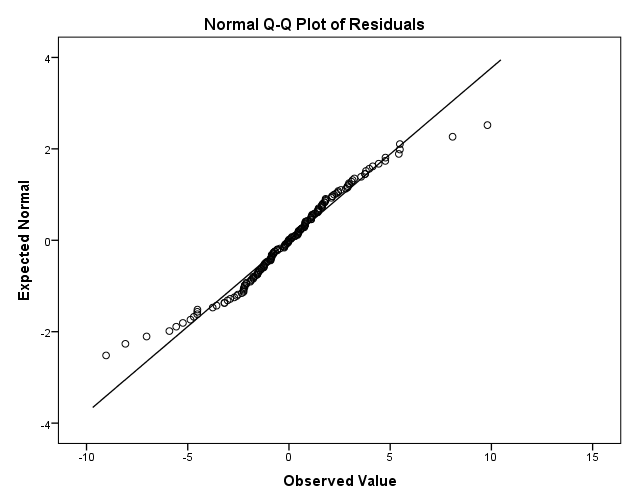


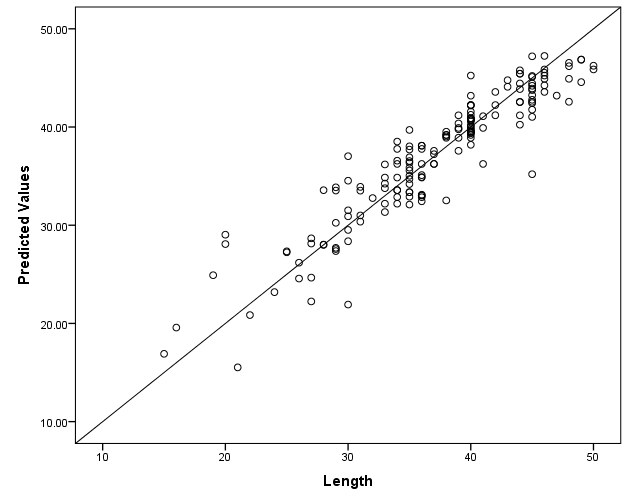


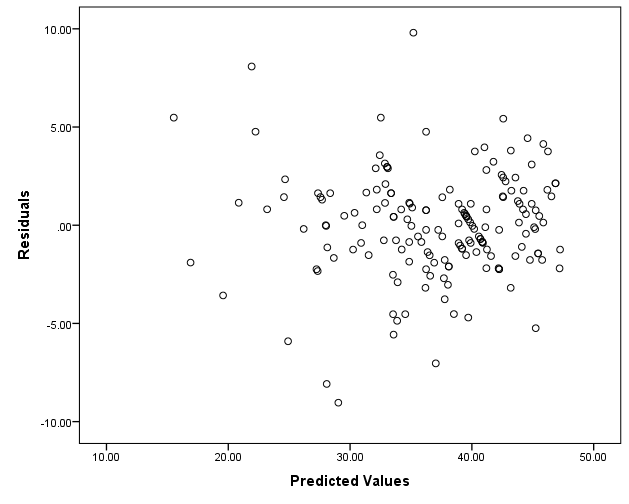

Supplement: Supplementary file 1 — Supplementary material 1 (DOCX 145 kb) [file 13197_2019_3803_MOESM1_ESM.docx]
